# Comparison of Relative and Absolute Abundance and Biomass of Freshwater Phytoplankton Taxa Using Metabarcoding and Microscopy

**DOI:** 10.1002/ece3.70856

**Published:** 2025-03-19

**Authors:** Ivan S. Mikhailov, Yurij S. Bukin, Alena D. Firsova, Darya P. Petrova, Yelena V. Likhoshway

**Affiliations:** ^1^ Limnological Institute Siberian Branch of the Russian Academy of Sciences Irkutsk Russia

**Keywords:** 18S rRNA, metabarcoding, microscopy, phytoplankton

## Abstract

Phytoplankton is the basis of the food web and an indicator of environmental change in aquatic ecosystems. Phytoplankton assessment uses microscopy, which estimates the composition, absolute abundance and biomass of taxa, and metabarcoding, which estimates the composition, high richness and diversity, and relative abundance of taxa. Problems remain with the consistency of results from these two methods and the quantification of metabarcoding. Using 18S rRNA metabarcoding and microscopy we compared the relative or absolute abundance and biomass of phytoplankton taxa (class or genus/species) in the south basin of Lake Baikal in spring over 3 years. Absolute abundance/biomass of phytoplankton taxa estimated by metabarcoding was obtained by combining relative abundances of amplicon sequence variants (ASV produced by error‐correcting method) derived from the V8–V9 region of 18S rRNA gene amplicon sequencing (primers were used that accurately represented the mean relative abundance of different microalgae) with total or class‐specific abundance/biomass of phytoplankton estimated by light microscopy. Many Spearman correlations were found between relative (non‐ or clr‐transformed) or absolute abundances/biomasses of the same phytoplankton classes or genus/species. Correlation coefficients were higher between absolute values than between relative values. Correlations were found between relative or absolute abundance/biomass, estimated by both methods, of the classes Bacillariophyceae, Coscinodiscophyceae, Mediophyceae, Chrysophyceae, Cryptophyceae, and Chlorophyceae, but not Dinophyceae and Trebouxiophyceae. Correlations were found between relative or absolute abundance/biomass of dominant species and ASVs of diatoms (*Ulnaria*, *Aulacoseira*, *Stephanodiscus*), Chrysophyceae (*Dinobryon*), and Cryptophyceae (*Cryptomonas*). Thus, the consistency of the dynamics of the relative or absolute abundance/biomass of phytoplankton taxa estimated by the two methods was revealed. Absolute abundances/biomasses of taxa estimated by metabarcoding in combination with microscopy improve the accuracy of metabarcoding‐based ecological assessment.

## Graphical abstract

In this study, 18S rRNA metabarcoding and microscopy revealed that the dynamics of relative and absolute abundance/biomass of phytoplankton taxa (classes, genera/species) are consistent in the southern basin of Lake Baikal in spring over 3 years. Absolute abundance/biomass of taxa (ASV, amplicon sequence variants) was obtained by combining relative abundances of phytoplankton taxa (ASV) with total or class‐specific abundance/biomass of phytoplankton estimated by light microscopy. The use of quantification increased the consistency in the dynamics at the class and genera/species between metabarcoding and microscopy data.

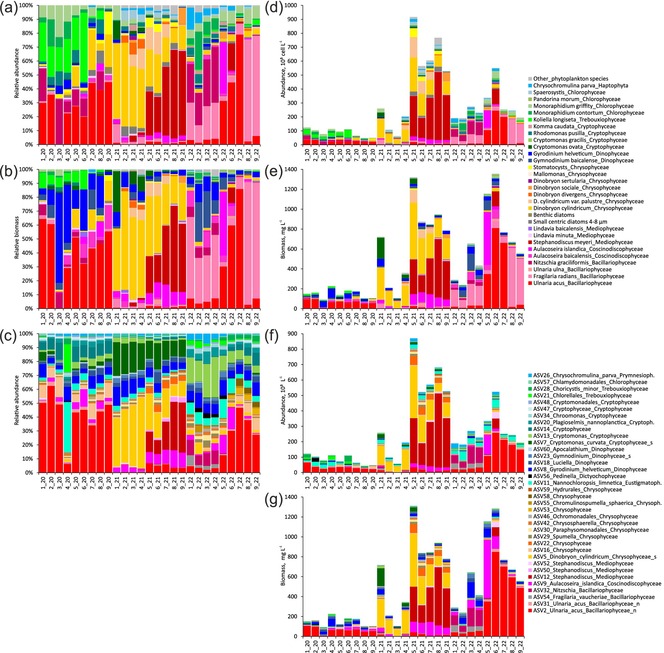

## 1 Introduction

Phytoplankton composition, abundance and biovolume are useful in determining the ecological status of aquatic ecosystems. Phytoplankton responds rapidly to environmental changes, making it a sensitive indicator of anthropogenic biogenic loading and climate change (Barton et al. 2016).

Microscopy is commonly used to identify and quantify phytoplankton. The advantages of light microscopy include the assessment of cell morphology (size, shape, life stages), mode of existence (solitary, colonial) and visible features such as pigment colors, some features of cell ultrastructure (stigmas, pyrenoids, chloroplasts), which are used to determine species composition, and the counting of abundance, biovolume or biomass (converted from biovolume estimates based on cell shape) of phytoplankton taxa. The disadvantages of light microscopy are the inability to detect phytoplankton destroyed during fixation, distinguish morphologically similar (cryptic) species, and identify small species due to insufficient optical resolution. Cryptic and small species of diatoms (Amato et al. 2007), chrysophytes (Škaloud, Kristiansen, and Škaloudová 2013), and green (Fawley, Fawley, and Hegewald 2011) are identified using scanning electron microscopy (SEM) and transmission electron microscopy (TEM). Instead of light microscopy, flow cytometry can be used to count phytoplankton cells (Marie, Simon, and Vaulot 2005). Quantitative PCR (qPCR) and fluorescence in situ hybridization (FISH) are also used to detect and enumerate phytoplankton (Karlson, Cusack, and Bresnan 2010).

The use of DNA metabarcoding based on high‐throughput sequencing (HTS) of DNA marker genes has advantages such as the most comprehensive assessment of the structure, richness, and diversity of microeukaryotes including phytoplankton (De Vargas et al. 2015; Xue et al. 2018; Burki, Sandin, and Jamy 2021). The disadvantages of metabarcoding are the assessment of the relative abundance of taxa and the many uncertainties that affect its results. The composition and relative abundance of taxa estimated using metabarcoding are affected by biological biases (gene copy number variation, tissue cell density, and cell biovolume), technical biases (DNA extraction, PCR amplification), and biases associated with HTS (library construction, HTS technology, and bioinformatics processing; Tan et al. 2015; Vasselon et al. 2018; Shelton et al. 2023). The identification of taxa by metabarcoding depends on the completeness of reference databases. To improve metabarcoding results, careful selection of primers that accurately estimate target organisms and their relative abundances (Bradley, Pinto, and Guest 2016; Latz et al. 2022) is used, as well as bioinformatic methods to correct for amplicon sequencing errors caused by PCR and sequencing (Eren et al. 2015; Callahan et al. 2016; Edgar 2016) that infer accurate biological sequences from noisy reads.

Quantification of metabarcoding is one of the current problems in the field of environmental DNA and microbiome research (Vasselon et al. 2018; Shelton et al. 2023; Blackman et al. 2024). Quantification is necessary because identical relative abundances of different taxa may not reflect the same absolute abundances. Use of synthetic DNA spike‐in in metabarcoding facilitate to quantify absolute microbial abundances (Tourlousse et al. 2017; Tkacz, Hortala, and Poole 2018). Quantitative PCR together with metabarcoding was used to estimate the absolute abundance of bacterial taxa (Jian et al. 2020). Taxa‐specific 18S rRNA gene copy number correction can be used for quantitative metabarcoding of microeukaryotes including phytoplankton, but information on gene copy number of different species remains limited (Gong and Marchetti 2019; Martin et al. 2022). To improve the quantification of diatoms using metabarcoding, a species‐specific correction factor (CF) based on cell biovolume, which correlates with 18S rDNA (Godhe et al. 2008) and rbcL (Vasselon et al. 2018) copy number, can be used.

Metabarcoding and microscopy have been used together to better characterize the composition, richness, and diversity of microeukaryotic communities and to assess their seasonal dynamics (Gran‐Stadniczeñko et al. 2019; Santi et al. 2021; Caracciolo et al. 2022; Mikhailov et al. 2022). Comparative studies of metabarcoding and microscopy for phytoplankton characterization show how consistent their results are and highlight the strengths and weaknesses of each method. Metabarcoding reveals higher phytoplankton species richness by identifying small, cryptic, and rare species than light microscopy (Xiao et al. 2014; Gran‐Stadniczeñko et al. 2019; Huo et al. 2020; Šimunović et al. 2023). Comparing the relative abundance of phytoplankton taxa estimated by metabarcoding with the relative abundance or biomass of phytoplankton taxa estimated by microscopy allows us to test which of the quantitative metrics estimated by these methods are in better agreement with each other (Obertegger, Pindo, and Flaim 2021; Andersson et al. 2023). A correlation was found between the relative abundance of phytoplankton species belonging to Bacillariophyta and Haptophyta estimated by metabarcoding and microscopy in large alpine lakes (Gelis et al. 2024).

However, correlations between the relative abundance of individual phytoplankton taxa estimated by metabarcoding and microscopy and the relationship of these results to the absolute abundances and biomasses of the same phytoplankton taxa remain poorly understood.

The aim of the work was to compare relative and absolute abundance and biomass of freshwater phytoplankton taxa using metabarcoding and microscopy. The objectives of this study were to compare the relative abundance/biomass of phytoplankton taxa (classes, genera/species) estimated by metabarcoding and microscopy; to calculate the absolute abundance/biomass of phytoplankton taxa using metabarcoding combined with microscopy; to compare the absolute abundance/biomass of phytoplankton taxa based on both methods; and to compare correlations obtained on relative and absolute abundances/biomasses of phytoplankton taxa. For this aim, water samples from the largest freshwater lake, the south basin of Lake Baikal were collected in spring (homothermy and mixing period) over 3 years and analyzed by microscopy (light microscopy to determine phytoplankton species composition, abundance, and biomass; scanning electron microscopy to identify of cryptic and small species) and metabarcoding (V8–V9 region of the 18S rRNA gene amplified using eukaryotic primers, which accurately represents the mean relative abundance of microalgal species belonging to different classes).

## 2 Methods

### 2.1 Study Area and Sampling

**FIGURE A1:**
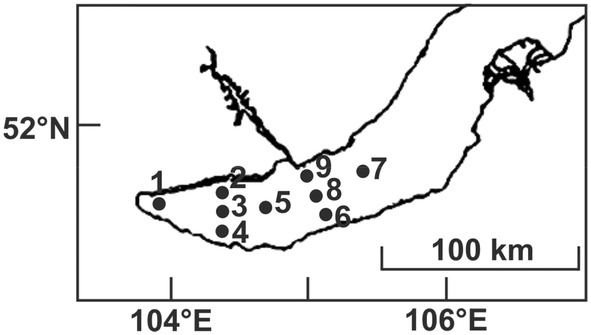
Map of sampling stations in the southern basin of Lake Baikal.

Integral water samples were collected from the RV “G. Yu. Vereshchagin” with an SBE 32 Carousel water sampler (Sea‐Bird Electronics, USA) into 1.5‐L polyethylene bottles from the upper 0.5, 5, 10, 15, 20, and 25 m layers at 9 stations of Lake Baikal from 1 June to 3 June 2020; from 2 June to 4 June 2021; and from 2 June to 4 June 2022 (Table A1 and Figure A1: Appendix A).

To collect microbial biomass, integral water samples of 10 L (equal volumes of samples from 0.5 to 25 m depth) were prefiltered with a 100‐μm nylon mesh, and then filtered through a 0.2‐μm analytical track membrane (Reatrek, Russia) using an AF‐142 filtration apparatus (Vladisart, Russia) with a compressor and a 20 L stainless steel pressure vessel. The biomass was washed off the filters into sterile TE buffer (10 mM Tris–HCl, 1 mM EDTA, pH 8.0) and stored at −80°C until analysis.

### 2.2 Microscopy

For phytoplankton analysis by light microscopy, 1.2 L of each integrated sample fixed with Lugol's solution was concentrated by settling and siphoning. Phytoplankton cells were counted by the Hensen method (Kiselev 1969) on a lined glass slide using an Axiostar Plus microscope (Zeiss, Oberkochen, Germany) equipped with a TOUPCAM UA1000CA camera (ToupTek Photonics, Hangzhou, China). The species composition of the phytoplankton was determined by morphological and other visible characteristics. Cell volume was calculated by measuring morphometric characteristics (diameter, length, and width) and geometric shape (Makarova and Pichkily 1970). The mass of cells of a given species is calculated by multiplying the average volume of its cells by the density of phytoplankton, which is equal to one. The biomass of a given species is calculated by multiplying the number of cells by their mass (Belykh et al. 2011). Cyanobacteria were counted by light microscopy but cannot be detected by 18S rRNA, so we excluded them from all analyses.

For scanning electron microscopy (SEM), 20 mL of each unfixed integrated sample was precipitated in the field onto filters (diameter 13 mm, 0.8 μm pores; Whatman, USA) using a syringe equipped with a special nozzle. 20 mL of 70% ethanol was then pumped through the filters, which were then attached to SEM stubs with double‐sided tape and dried. They were then coated with gold in the laboratory using an SCD vacuum evaporator (Blazers Union Ltd., Balzers, Liechtenstein) and analyzed using a Quanta 200 SEM (FEI Electron Optics B.V., Eindhoven, The Netherlands). To determine the relative abundance of the morphologically similar species *Ulnaria acus* (Kützing) Aboal and *Fragilaria radians* (Kützing) D.M. Williams and Round, a minimum of 100 cells were counted on a filter in each sample using SEM. The absolute abundance and biomass of these species were calculated by multiplying the relative abundance of each of the two species by the total abundance of these species, estimated by light microscopy.

### 2.3 DNA Extraction, PCR Amplification, and Sequencing

DNA was extracted using lysozyme, proteinase K, SDS, and phenol/chloroform (Rusch et al. 2007) with modifications (Bukin et al. 2023). The V8‐V9 region of the 18S rRNA gene was amplified using V8f (5′‐ATAACAGGTCTGTGATGCCCT‐3′) and 1510R (5′‐CCTTCYGCAGGTTCACCTAC‐3′) eukaryotic primers, which accurately represent mean relative abundance of freshwater and marine microalgal (phytoplankton) species (Bradley, Pinto, and Guest 2016). Previously, these primers were tested on seven microalgal mock communities (12 algal species belonging to the classes Bacillariophyceae, Trebouxiophyceae, Chlorophyceae, Cryptophyceae, Dinophyceae, Prymnesiophyceae, Coccolithophyceae, Chrysophyceae, Eustigmatophyceae) with different relative abundances and then on environmental samples (freshwater, coastal, and wastewater) for validation. These V8–V9 primers showed consistently low sequencing errors, that is, the percentage difference between mock community sequence reads (sequenced by Illumina MiSeq) and their known Sanger sequences (0.01% on average for processed sequences; Bradley, Pinto, and Guest 2016). For the V8–V9 of the 18S rRNA, the conditions were 95°C for 3 min, followed by 25 cycles of 98°C for 20 s, 65°C for 15 s, and 72°C for 15 s, with a final extension at 72°C for 10 min (Bradley, Pinto, and Guest 2016). Amplicon library preparation was performed as described in “16S Metagenomic Sequencing Library Preparation. Preparing 16S Ribosomal RNA Gene Amplicons for the Illumina MiSeq System #15044223 Rev. B.” Amplicon libraries were sequenced on the MiSeq sequencer (2 × 300 bp, Illumina) using the MiSeq Reagent Kit v3 at the Smorodintsev Research Institute of Influenza (National Influenza Centre of Russia; Saint Petersburg).

### 2.4 Sequence Data Processing

Analysis of reads was performed in DADA2 v1.16 (R‐package *dada2*) to generate ASV (Callahan et al. 2016). Paired‐end reads from 18S rRNA gene regions were filtered, merged and chimeras were removed according to the tutorial for the R‐package *dada2*. The *filterAndTrim()* function had the following parameters: trimLeft = c(21, 20), truncLen = c(250, 165), maxN = 0, maxEE = c(2, 3), truncQ = 2. The remaining parameters were default values. Taxonomic assignment was performed using the naive Bayesian classifier implemented in the R‐package *dada2* (*assignTaxonomy()* function). PR2 version 5.0.0 (Guillou et al. 2013), Silva Eukaryotic 18S version 132 (Quast et al. 2012), and NCBI were used for taxonomic assignment of microeukaryotes. ASVs with ≤ 2 reads, and ASVs classified as Metazoa, Streptophyta, Nucleomorph (:nucl), Bacteria, Archaea and unclassified at the domain level were removed.

**FIGURE B1:**
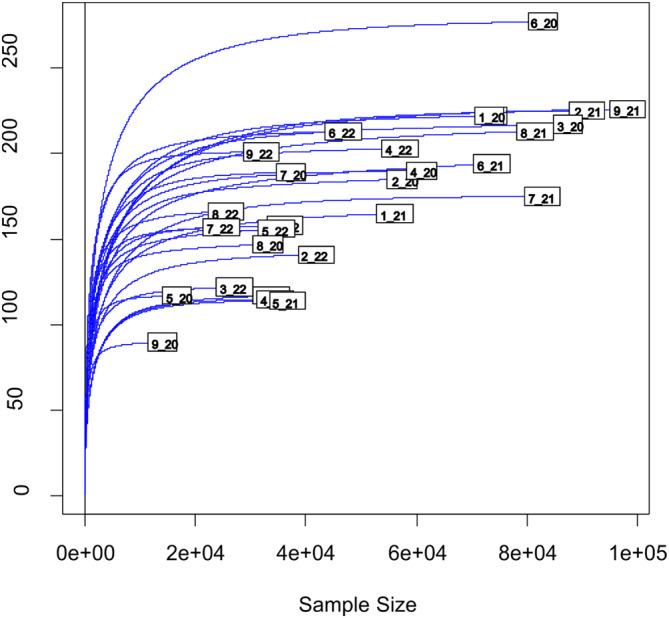
Rarefaction curves of microeukaryotic 18S rRNA reads.

The average length and number of reads in the samples were 322 bp and 13,993–98,176 (Table B1 and Figure B1: Appendix B), respectively. The species richness in all samples was 1003 ASVs. The rarefaction curves calculated for all samples reached saturation (Table B1 and Figure B1: Appendix B), indicating that the number of reads was sufficient to assess species richness. For all samples, Chao1 has a similar value to richness, indicating that we have estimated the full ASV richness. The species richness was 90–277 ASVs, the Shannon index was 2.32–3.91, and the Simpson index was 0.74–0.96 (Table B1 and Figure B1: Appendix B).

### 2.5 Statistical Analysis

Species richness, Chao1 (O'Hara 2005), Shannon and Simpson indices of biodiversity (Hill 1973) based on the abundance of phytoplankton species and the number of ASVs reads were calculated using the R‐package *vegan* (Oksanen et al. 2019). R‐package *vegan* was used to construct rarefaction curves based on the number of ASVs reads.

**FIGURE C1:**
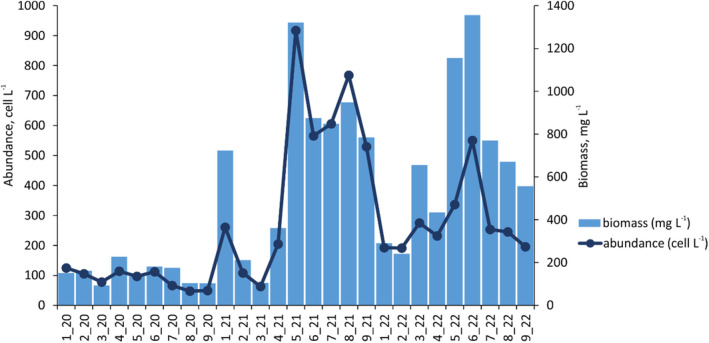
Total phytoplankton abundance and biomass based on light microscopy.

For statistical analysis, quantitative data of species based on microscopy (absolute abundance [×10^3^ cell L^−1^] and biomass [mg L^−1^], and relative abundance and biomass), ASV (number of reads converted to relative abundance of reads) and ASV combined with microscopy data (absolute abundance/biomass of ASV combined with total or class‐specific phytoplankton abundance/biomass) were used. We assume that the relative abundance of phytoplankton ASV (taxon) reads per sample corresponds to the relative abundance of that ASV (taxon) in the total phytoplankton abundance/biomass estimated by microscopy. Therefore, the absolute abundance/biomass of phytoplankton ASVs (ASV × total value) was calculated by multiplying the relative abundance of these ASVs by the sample‐specific total phytoplankton abundance (cell L^−1^) and biomass (mg L^−1^) based on microscopy (Figure C1: Appendix C). Calculations were performed using a script in R. R is the relative abundance of reads of a given ASV (or zOTU) produced by error‐correcting methods. The absolute abundance of the ASV is calculated using the following formulas:

(1) $$A_{j}=\sum_{i=1}^{n_{j}}a_{\mathrm{ij}}$$

where $A_{j}$ is the total phytoplankton abundance in sample *j*, $n_{j}$ is the number of phytoplankton species in sample *j*, $a_{\mathrm{ij}}$ is the absolute abundance of the *i* phytoplankton species in sample *j*.

(2) $${\bar{a}}_{\mathrm{ij}}=R_{\mathrm{ij}}A_{j}$$

where ${\bar{a}}_{\mathrm{ij}}$ is the calculated absolute abundance of *i* ASV phytoplankton in sample *j*, $R_{\mathrm{ij}}$ is the relative abundance of *i* ASV in sample *j*.

The absolute biomass of ASV is calculated using the following formulas:

(3) $$M_{j}=\sum_{i=1}^{n_{j}}m_{\mathrm{ij}}$$

where $M_{j}$ is the total phytoplankton biomass in sample *j*, $n_{j}$ is the number of phytoplankton species in sample *j*, $m_{\mathrm{ij}}$ is the absolute biomass of the *i* phytoplankton species in sample *j*.

(4) $${\bar{m}}_{\mathrm{ij}}=R_{\mathrm{ij}}M_{j}$$

where ${\bar{m}}_{\mathrm{ij}}$ is the calculated absolute biomass of *i* ASV phytoplankton in sample *j*, $R_{\mathrm{ij}}$ is the relative abundance of *i* ASV in sample *j*.

It is assumed that a more accurate absolute abundance/biomass of phytoplankton ASVs (ASV × class‐specific value) was calculated by multiplying the class‐specific relative abundance of ASVs by the sample‐ and class‐specific phytoplankton abundance (cell L^−1^) and biomass (mg L^−1^) based on microscopy. Calculations were performed using a script in R. The absolute abundance of ASV is calculated using the following formulas:

(5) $$A_{\mathrm{jc}}=\sum_{i=1}^{n_{\mathrm{jc}}}a_{\mathrm{ijc}}$$

where $A_{\mathrm{jc}}$ is the absolute abundance of class *c* phytoplankton in sample *j*, $n_{jc}$ is the number of class *c* species in sample *j*, $a_{\mathrm{ijc}}$ is the absolute abundance of class *c* phytoplankton species *i* in sample *j*.

(6) $${\bar{a}}_{\mathrm{ijc}}=R_{\mathrm{ijc}}A_{\mathrm{jc}}$$

where ${\bar{a}}_{\mathrm{ijc}}$ is the calculated absolute abundance of *i* ASV of class *c* in sample *j*, $R_{\mathrm{ijc}}$ is the relative abundance of *i* ASV from the total number of reads of class *c* in sample *j*.

The absolute biomass of ASV is calculated using the following formulas:

(7) $$M_{\mathrm{jc}}=\sum_{i=1}^{n_{\mathrm{jc}}}m_{\mathrm{ijc}}$$

where $M_{\mathrm{jc}}$ is the absolute biomass of class *c* phytoplankton in sample *j*, $n_{\mathrm{jc}}$ is the number of class *c* species in sample *j*, $m_{\mathrm{ijc}}$ is the absolute biomass of class *c* phytoplankton species *i* in sample *j*.

(8) $${\bar{m}}_{\mathrm{ijc}}=R_{\mathrm{ijc}}M_{\mathrm{jc}}$$

where ${\bar{m}}_{\mathrm{ijc}}$ is the calculated absolute biomass of *i* ASV belonging to class *c* in sample *j*, $R_{\mathrm{ijc}}$ is the relative abundance of *i* ASV from the total number of reads of class *c* in sample *j*.

To avoid compositionality, the relative values were transformed before correlation analysis using the centered log‐ratio (clr) transformation (Aitchison 1986) using the R‐package *compositions* (Van den Boogaart and Tolosana‐Delgado 2008).

The relationships between different quantitative taxonomic data of phytoplankton (relative, clr‐transformed relative or absolute abundance/biomass), ASV (relative, clr‐transformed relative or absolute abundance/biomass) were assessed by coinertia analysis (CIA) using the R‐package *ade4* (Dray and Dufour 2007) based on PCoA analyses. In CIA, the correlation (*R*) of multivariate vectors (*V*) indicates the variability between the two multivariate quantitative data matrices.

The relationship between species richness of different phytoplankton classes estimated by microscopy (number of species) and by metabarcoding (number of ASV) was assessed using a correlation analysis based on Spearman's *r* correlation coefficient (Hollander and Wolfe 1973). Pairwise relationships between phytoplankton classes or species/ASV, based on relative/absolute and abundance/biomass, were assessed using Spearman's *r* correlation coefficient. Species and ASVs present in eight or more samples were used for correlation analyses. The accuracy of the correlation coefficients was determined using ‘W’ Spearman statistics (*p* value ≤ 0.05; Royston 1995). The *p* values of Spearman correlation coefficients were corrected for the false discovery rate in multiple comparisons using the Benjamini‐Hochberg equation (Benjamini and Hochberg 1995). Correlation coefficients were aggregated into a matrix, which was visualized by a heat map generated using R‐package *gplots* (Warnes et al. 2015). Rows and columns in the correlation matrix were clustered using R‐package *gplots* (Euclidean distance metric and the complete‐link clustering method).

## 3 Results

### 3.1 Comparison of Phytoplankton Structure and Species Richness Estimated by Microscopy and Metabarcoding

**FIGURE 1:**
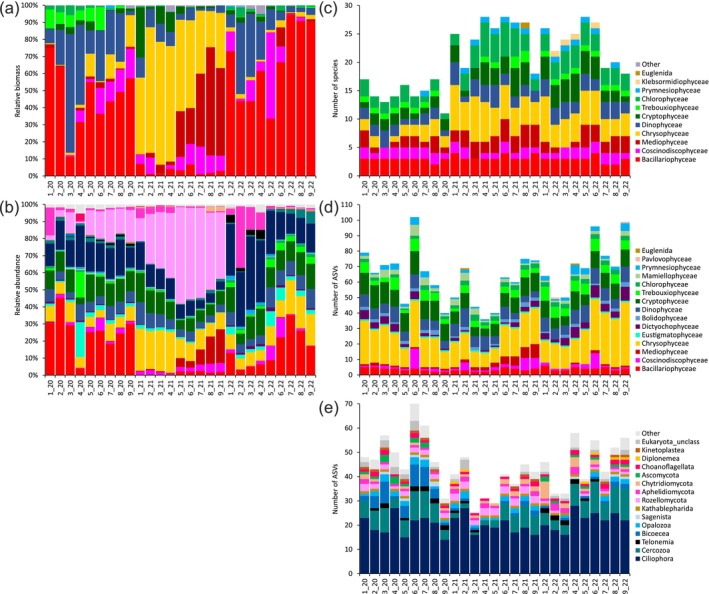
Relative biomass of phytoplankton classes (a). Relative abundance of high‐rank microeukaryotic taxa based on read counts (b). Species richness of phytoplankton classes based on microscopy (c) and ASVs (d), and high‐rank taxa of heterotrophic microeukaryotes based on ASVs (e). Names of sampling sites 1–9. Sampling years _20‐2020, _21‐2021, _22‐2022.

Using microscopy, 11 phytoplankton classes were identified (Figure 1a,c). Based on relative biomass, the Bacillariophyceae class dominated in 2020 and 2022, while Chrysophyceae and Mediophyceae dominated in 2021 (Figure 1a). The relative biomass of Dinophyceae was high at several stations in 2020 and 2022. Phytoplankton richness was assessed for all species identified using light and scanning electron microscopy. Among the phytoplankton classes, Chrysophyceae (up to eight species per sample), Chlorophyceae (1–8 species), Bacillariophyceae (2–4 species), and Cryptophyceae (up to five species) had high richness (Figure 1c).

Using metabarcoding according to PR2 taxonomy, 7 supergroups, 16 divisions, and 66 classes of microeukaryotes were identified. Based on relative abundance, Bacillariophyceae (3%–45%) and Ciliophora (11%–43%) dominated in 2020 and 2022, whereas Chrysophyceae (7%–24%), Mediophyceae (0.3%–20%), and Rozellomycota (13%–56%) dominated in 2021 (Figure 1b). The relative abundances of Cryptophyceae (4%–17%) and Dinophyceae (3.5%–13%) were similar in all years. The proportion of Aphelidiomycota was high in 1_20 (16%) and 2–4_22 (10%–35%). Microeukaryotic richness was estimated for 463 ASVs, representing 99% of the total number of reads in the samples. Among the 15 phytoplankton classes identified, Chrysophyceae (9–41 ASVs per sample), Cryptophyceae (5–15 ASVs), Dinophyceae (4–12 ASVs), Trebouxiophyceae (2–9 ASVs), and Bacillariophyceae (2–6 ASVs) had high richness (Figure 1d). Among the high‐rank taxa of heterotrophic microeukaryotes, Ciliophora (14–28 ASVs), Cercozoa (1–13 ASVs), and Bicoecea (up to 9 ASVs) had high richness (Figure 1e).

Correlation analysis showed significant positive correlations between species number and ASVs only for the classes Mediophyceae (0.52, *p* = 0.005) and Chlorophyceae (0.49, *p* = 0.008; Tables D1, D2, D3: Appendix D). Shannon indices were calculated for phytoplankton species abundances (Tables D1, D2, D3: Appendix D) and biomasses and for the top 36 phytoplankton ASVs (Tables D1, D2, D3: Appendix D). Correlations were found between Shannon indices for phytoplankton species abundances and biomasses (0.77, *p*=5.7×10^‐5^) and between Shannon indices for species biomasses and phytoplankton ASVs (0.43, *p*=0.026).

### 3.2 Consistency Between Phytoplankton Structure Assessed by Microscopy and Metabarcoding

From the top 58 microeukaryotic ASVs (77%–95% of the total number of reads in the samples), 22 heterotrophic ASVs were removed to then compare 36 phytoplankton ASVs (autotrophs and mixotrophs) with phytoplankton species assessed by microscopy. The dominant 23 phytoplankton species and 1 phytoplankton group (stomatocysts) and the top 33 phytoplankton ASVs (species and ASVs present in 8 or more samples) were used in coinertia analysis to determine the relationship between community structure assessed by light microscopy (mic) and metabarcoding (meta). The RV coefficients in the coinertia analysis between absolute abundances or biomasses of species and ASV (ASV × total value) were higher than between their relative values (Table 1).

**TABLE 1:** RV coefficients in the co‐inertia analysis of different quantitative data.

Сompared data	Relative values	Relative values transformed by ranking from 0 to 1	Clr‐transformed relative values	Absolute values
ASV abundance—species abundance	0.649	0.688	0.704	0.947
ASV abundance—species biomass	0.698	0.714	0.723	0.882^a^

^a^ ASV absolute biomass/species absolute biomass.

**FIGURE 2:**
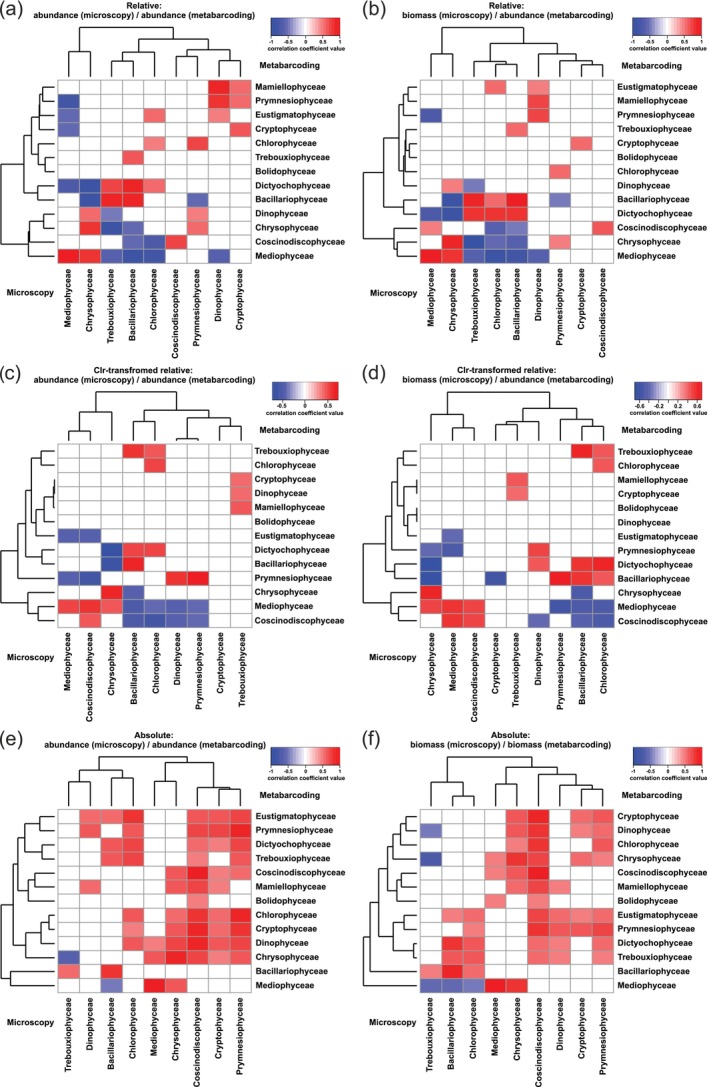
Heat maps showing correlations between phytoplankton classes with different quantitative metrics estimated by microscopy (mic) and metabarcoding (meta): (a) relative abundance (mic) with abundance (meta); (b) relative biomass (mic) with abundance (meta); (c) clr‐transformed relative abundance (mic) with abundance (meta); (d) clr‐transformed relative biomass (mic) with abundance (meta); (e) absolute abundance (mic) with abundance (meta); (f) absolute biomass (mic) with biomass (meta). Colors indicate the *r*‐values of Spearman's correlation coefficients. The reliable value (*p* ≤ 0.05) of the correlation coefficient is *r* ≥ |0.38|.

Correlations were found between phytoplankton classes assessed by light microscopy (23 species and 1 group) and metabarcoding (all phytoplankton ASVs) with different quantitative metrics (Figure 2).

Relative abundance (mic)/abundance (meta) pairs (16 negative and 22 positive; Figure 2a) and biomass (mic)/abundance (meta) pairs (15 negative and 20 positive) had a similar number of correlations (Figure 2b). The clr‐transformed relative abundance (mic)/abundance (meta) pairs (15 negative and 16 positive; Figure 2c) and biomass (mic)/abundance (meta) pairs (13 negative and 18 positive; Figure 2d) had slightly lower correlations than the relative values. The absolute abundance (mic)/ abundance (meta) pairs (2 negative and 54 positive; Figure 2e) and biomass (mic)/ biomass (meta) pairs (5 negative and 50 positive; Figure 2f) had more correlations than the relative values. The mic/meta pairs for Bacillariophyceae, Coscinodiscophyceae, Mediophyceae, Chrysophyceae, and Cryptophyceae showed high correlation coefficients for all metrics (highest for absolute values; Table 2). Chlorophyceae showed correlations mainly only for abundances (relative and absolute). Prymnesiophyceae had correlations only for clr‐transformed and absolute abundance/biomass. High correlation coefficients were also found for several classes (Table 2). Thus, the total number of correlations (mostly positive; Figure 2) and their coefficients (Table 2) were higher for absolute values than for relative values.

**TABLE 2:** Spearman correlation coefficients between phytoplankton classes with different quantitative metrics based on microscopy (mic) and metabarcoding (meta).

Pairs of correlated classes: microscopy (mic)/metabarcoding (meta)	Relative values				Absolute values	
	Abundance (mic)/abundance (meta)	Biomass (mic)/abundance (meta)	Clr‐transformed		Abundance (mic)/abundance (meta × total phyto. abundance)	Biomass (mic)/biomass (meta × total phyto. biomass)
			Abundance (mic)/abundance (meta)	Biomass (mic)/abundance (meta)		
Bacillariophyceae_mic/Bacillariophyceae_meta	0.66	0.74	0.64	0.57	0.68	0.77
Coscinodiscophyceae_mic/Coscinodiscophyceae_meta	0.56	0.53	0.46	0.5	0.83	0.83
Mediophyceae_mic/Mediophyceae_meta	0.78	0.8	0.5	0.54	0.88	0.87
Chrysophyceae_mic/Chrysophyceae_meta	0.64	0.68	0.63	0.62	0.76	0.7
Dinophyceae_mic/Dinophyceae_meta	—	—	—	—	—	—
Cryptophyceae_mic/Cryptophyceae_meta	0.51	0.42	—	—	0.51	0.51
Trebouxiophyceae_mic/Trebouxiophyceae_meta	—	—	—	—	—	—
Chlorophyceae_mic/Chlorophyceae_meta	0.39	—	0.53	0.46	0.6	—
Prymnesiophyceae_mic/Prymnesiophyceae_meta	—	—	0.67	—	0.75	0.64
Trebouxiophyceae_mic/Dictyochophyceae_meta	0.58	0.64	—	—	—	—
Trebouxiophyceae_mic/Mamiellophyceae_meta	—	—	0.44	0.42	—	—
Chlorophyceae_mic/Dictyochophyceae_meta	0.47	0.65	0.52	0.64	0.62	0.58
Chlorophyceae_mic/Eustigmatophyceae_meta	0.43	0.45	—	—	0.7	0.49

### 3.3 Comparison of Relative or Absolute Abundance and Biomass of Phytoplankton Species and ASV

**FIGURE 3:**
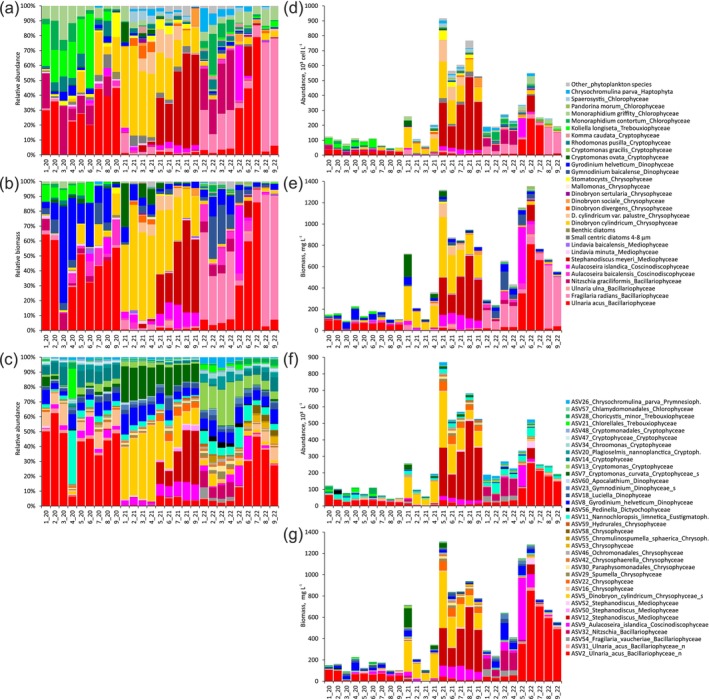
Relative abundance (a) and biomass (b) of phytoplankton species. Relative abundance of the top 36 phytoplankton ASVs based on read counts (c). Absolute abundance (d) and biomass (e) of phytoplankton species. Absolute abundance (f) and biomass (g) of the top 33 phytoplankton ASVs (ASV × class‐specific value). _n—ncbi; _s—silva.

According to microscopy, the spatiotemporal dynamics of relative abundance (Figure 3a) and biomass (Figure 3b) or absolute abundance (Figure 3d) and biomass (Figure 3e) of dominant species were similar, but differed for small‐ and large‐cell phytoplankton species. For example, the small‐cell green microalga *Koliella longiseta* (Vischer) Hindák (2020) and the diatom *Nitzschia graciliformis* Lange‐Bertalot & Simonsen (2020, 2022) had high abundance (up to 55 × 10^3^ cell L^−1^ and up to 92 × 10^3^ cell L^−1^, respectively; Figure 3a,d) and low biomass (up to 24 mg L^−1^ and up to 37 mg L^−1^, respectively; Figure 3b,e). The large‐cell dinophytes *Gymnodinium baicalense* N.L. Antipova and *Gyrodinium helveticum* (Penard) Y. Takano & T. Horiguchi had low abundance (up to 6.9 × 10^3^ cell L^−1^ and up to 7.3 × 10^3^ cell L^−1^, respectively; Figure 3a,d) and high biomass (up to 172 mg L^−1^ and up to 94 mg L^−1^, respectively; Figure 3b,e).

The spatiotemporal dynamics of the relative biomass of the dominant phytoplankton species (Figure 3b) and the relative abundance of their related ASVs (Figure 3c) were similar. Absolute abundance (Figure 3f) and biomass (Figure 3g) of the top 33 ASVs belonging to specific classes were calculated based on class‐specific values of the same phytoplankton classes estimated by microscopy. The relative (Figure 3b) or absolute (Figure 3e) biomass of phytoplankton species was compared with the relative abundance (Figure 3c) or absolute biomass (Figure 3g) of their related ASVs.

Microscopy revealed that the diatom *Ulnaria acus* was dominant in 2020 (up to 64%, up to 98 mg L^−1^) and at stations 6, 7 in 2022 (3%–60%, 8–810 mg L^−1^; Figure 3b,e). The diatom *Fragilaria radians* was dominant at stations 1–4, 8, 9 in 2022 (3%–88%, 38–591 mg L^−1^). The chrysophyte *Dinobryon cylindricum* O.E. Imhof (20%–59%, 62–563 mg L^−1^) and the diatom *Stephanodiscus meyeri* Genkal & Popovskaya (up to 62%, up to 587 mg L^−1^) dominated in 2021 (Figure 3b,e). Metabarcoding also showed that the diatom ASV2_Ulnaria_acus dominated in 2020 (6%–62%, 10–108 mg L^−1^) and several stations in 2022 (2%–49%, 29–849 mg L^−1^), and the chrysophyte ASV5_Dinobryon_cylindricum (8%–51%, 67–530 mg L^−1^) and the diatom ASV12_Stephanodiscus (up to 36%, up to 584 mg L^−1^) dominated in 2021 (Figure 3c,g).

For some related species and ASVs, the spatio‐temporal dynamics were partly similar. The diatom *Aulacoseira baicalensis* (K.I. Meyer) Simonsen had high biomass at some stations in 2020 (up to 17%, up to 20 mg L^−1^) and 2022 (up to 11%, up to 92 mg L^−1^). The diatom *Aulacoseira islandica* (O. Müller) Simonsen had high biomass at two stations in 2020 (st. 6%–5%, 9 mg L^−1^; st. 8%–11%, 11 mg L^−1^), 2021 (up to 16%, up to 136 mg L^−1^) and in 2022 at st. 5 (46%, 531 mg L^−1^; Figure 3b,e). ASV9_Aulacoseira_islandica also had high biomass at these two stations in 2020 (st. 6%–12%, 27 mg L^−1^; st. 8%–5%, 13 mg L^−1^), 2021 (1%–12%, up to 136 mg L^−1^) and at some stations in 2022 (st. 1, 4–6—3%–16%, up to 584 mg L^−1^; Figure 3c,g). The diatom *Nitzschia graciliformis* had a high relative biomass at stations 1, 3 in 2020 (up to 11%, up to 11 mg L^−1^) and at stations 1–4 in 2022 (5%–9%, 17–37 mg L^−1^; Figure 3b,e). ASV32_Nitzschia had high biomass at the same stations 1–4 in 2022 (7%–11%, 59–187 mg L^−1^; Figure 3c,g). The cryptophyte *Cryptomonas ovata* Ehrenberg had the highest biomass in 2021 at stations 1, 3–5 (4%–30%, 10–212 mg L^−1^; Figure 3b,e). *Cryptomonas gracilis* Skuja had the highest biomass in 2022 (up to 1%, up to 13 mg L^−1^). ASV7_Cryptomonas_curvata had the highest biomass at all stations in 2021 (11%–24%, up to 180 mg L^−1^; Figure 3c,g). ASV13_Cryptomonas had the highest biomass in 2022 (5%–29%, up to 10 mg L^−1^) and ASV14_Cryptophyceae was in 2020 and 2022 (0.04%–3%; Figure 3c,g). The dinophyte *Gyrodinium helveticum* had the highest relative biomass in 2020 (2%–64%) than in 2021 (1%–10%) and 2022 (0.5%–13%; Figure 3b). However, the absolute biomass was similar in these years (2–94 mg L^−1^; Figure 3e). The biomass of ASV8_Gyrodinium helveticum varied among years (3%–14%, 1–156 mg L^−1^; Figure 3с,3g). The prymnesiophyte *Chrysochromulina parva* Lackey had a low biomass (up to 2%, up to 3 mg L^−1^). ASV26_Chrysochromulina_parva had a higher relative abundance in 2022 at stations 1–4 (4%–7%) than in other years and stations (0.1%–2%) and its absolute biomass was low (up to 3 mg/L). The composition of species and ASVs and their spatiotemporal dynamics differed for Chlorophyta (Trebouxiophyceae, Chlorophyceae). *Koliella longiseta* had the highest biomass in 2020 at stations 1–6 (5%–13%, 6–24 mg L^−1^). *Monoraphidium contortum* (Thuret) Komárková‐Legnerová and *Monoraphidium griffithii* (Berkeley) Komárková‐Legnerová had the highest biomass in 2020 at stations 1–4 (0.6%–2.5%, 0.93–4 mg L^−1^; 1.3%–4.5%, 2–4 mg L^−1^, respectively) and at 2_22 (4%, 10 mg L^−1^; 1%, 2.7 mg L^−1^, respectively; Figure 3b,e). ASV21_Chlorellales had the highest biomass in 4_20 (20%, 10 mg L^−1^), and in 2022 (0.7%–4.9%, up to 0.5 mg L^−1^). The relative abundance of ASV28_Choricystis_minor was high in 4_20 (4.3%) and in 2022 (up to 2.7%; Figure 3b). The biomass of ASV28_Choricystis_minor was high in 2020 (up to 16 mg L^−1^; Figure 3g).

Eustigmatophyceae (Nannochloropsis_limnetica) and Dictyochophyceae (Pedinella) were identified by metabarcoding alone. Absolute abundance/biomass for them was calculated using class‐specific abundance/biomass of morphologically similar and correlated classes, for example, Nannochloropsis with Chlorophyceae, Pedinella with Trebouxiophyceae. ASV11_Nannochloropsis_limnetica had the highest abundance/biomass in 4_20 (37%, 26 × 10^3^ cell/L, 5.7 mg L^−1^), and in 2022 (4%–14%, 15–62 × 10^3^ cell L^−1^, 3.6–12 mg L^−1^; Figure 3c,f,g). ASV56_Pedinella had the highest relative abundance in 2022 (up to 3.6%) and the highest absolute abundance and biomass in 2020 (up to 23 × 10^3^ cell/L, up to 11 mg L^−1^).

### 3.4 Consistency Between Relative or Absolute Abundances and Biomasses of Phytoplankton Species and ASVs

**FIGURE 4:**
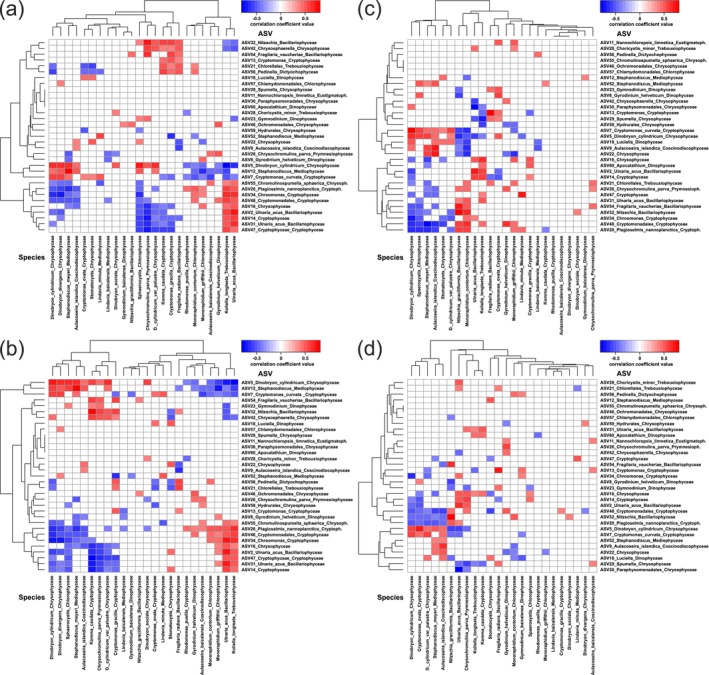
Heat maps showing correlations between species (mic) and ASV (meta) with relative values: (a) relative abundance (mic) and abundance (meta); (b) relative biomass (mic) and abundance (meta); (c) clr‐transformed relative abundance (mic) and abundance (meta); (d) clr‐transformed relative biomass (mic) and abundance (meta). Colors indicate the *r*‐values of Spearman's correlation coefficients. The reliable value (*p* ≤ 0.05) of the correlation coefficient is *r* ≥ |0.38|.

**FIGURE 5:**
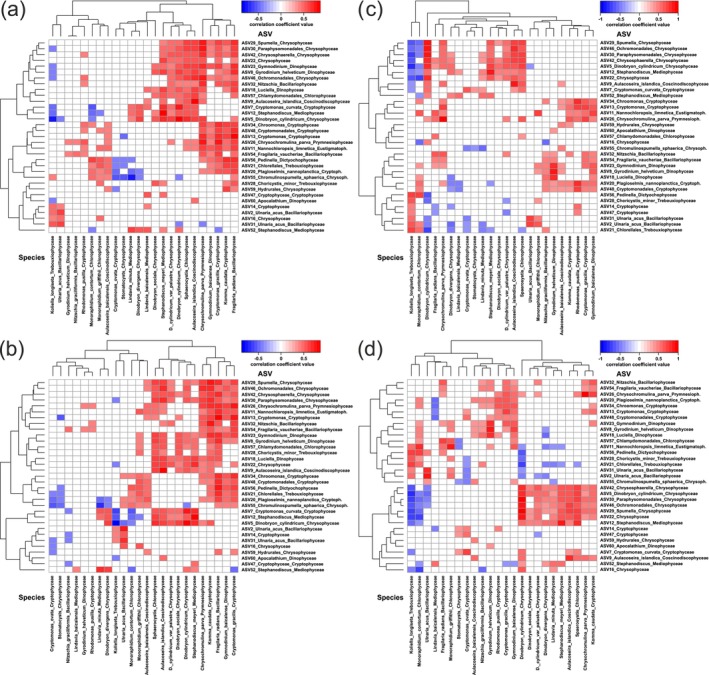
Heat maps showing correlations between species (mic) and ASV (meta) with absolute values: (a) abundance (mic) and abundance (meta: ASV × total value); (b) biomass (mic) and biomass (meta: ASV × total value); (c) abundance (mic) and abundance (meta: ASV × class‐specific value); (d) biomass (mic) and biomass (meta: ASV × class‐specific value). Colors indicate the *r*‐values of Spearman's correlation coefficients. The reliable value (*p* ≤ 0.05) of the correlation coefficient is *r* ≥ |0.38|.

For correlation analysis, relative (Figure 4a,b) and clr‐transformed relative (Figure 4c,d) abundances/biomasses of 23 species and one group and the top 33 ASVs were used. Absolute abundance/biomass of ASVs were calculated based on sample‐specific total phytoplankton abundance/biomass (ASV × total value; Figure 5a,b) or sample‐ and class‐specific phytoplankton abundance/biomass (ASV × class‐specific value; Figure 5c,d). The pairs of relative abundance (species)/abundance (ASV; 98 negative, 90 positive; Figure 4a) and biomass (species)/abundance (ASV; 101 negative, 93 positive; Figure 4b) had a similar number of correlations. The clr‐transformed relative abundance (species)/abundance (ASV) pairs (70 negative, 83 positive; Figure 4c) and biomass (species)/abundance (ASV) pairs (60 negative, 64 positive; Figure 4d) pairs had fewer correlations than the relative values. The absolute abundance (species)/abundance (ASV × total value; 32 negative, 235 positive; Figure 5a) and biomass (species)/biomass (ASV × total value; 33 negative, 234 positive; Figure 5b) pairs had significantly more correlations than the relative values. The absolute abundance (species)/abundance (ASV × class‐specific value; 47 negative, 177 positive; Figure 5c) and biomass (species)/biomass (ASV × class‐specific value; 56 negative, 190 positive; Figure 5d) pairs had fewer correlations than the absolute ASV × total value. Thus, there were more positive correlations between absolute values of species and ASVs than between relative values. The total number of correlations increased in the order clr‐transformed relative values, relative values, absolute ASV × class‐specific value, and absolute ASV × total value.

Correlations between dominant species and their related ASVs were common for relative and clr‐transformed relative and absolute abundance/biomass (Table E1: Appendix E). For low abundance species and ASVs, correlations were not present for all metrics. The highest correlation coefficients were generally for absolute abundance/biomass for ASV × class‐specific value, then slightly lower for ASV × total value, and the lowest were for relative abundance/biomass (Table E1: Appendix E).

## 4 Discussion

### 4.1 Сonsistency of Phytoplankton Structure Based on Microscopy and Metabarcoding

The combined use of metabarcoding and microscopy showed consistency in the results of these methods and improved the taxonomic resolution of the phytoplankton. Many of the phytoplankton classes were identified by both methods (Figure 1a,b) and correlations were found between the same classes based on different quantitative metrics (Figure 2). Phytoplankton richness was higher for ASVs than for species based on microscopy (Figure 1c,d). The classes Chrysophyceae, Cryptophyceae, Bacillariophyceae had higher species richness compared to other classes when determined by both methods. In all comparative studies of these methods, phytoplankton richness revealed by metabarcoding was higher than by microscopy (Rimet et al. 2018; Huo et al. 2020; Obertegger, Pindo, and Flaim 2021; Andersson et al. 2023).

In our work, microscopy identified the phytoplankton composition to species level, whereas metabarcoding identified it to species level and mostly to genera or high‐ranking taxa (Figure 3). This may be due to the lack of 18S rRNA sequences in the databases for some phytoplankton species identified by microscopy. Some species and genera identified by microscopy and metabarcoding coincided and had similar spatiotemporal dynamics (Figure 3) and high correlation coefficients between their relative abundances or biomasses (Figure 4, Table E1: Appendix E). Previously, k‐means clustering revealed that some phytoplankton OTUs (V8‐V9 18S rRNA) in Lake Baikal had similar seasonal dynamics to the biomasses of related phytoplankton species estimated by microscopy (Mikhailov et al. 2022). Microscopy was more effective than high‐throughput sequencing in identifying phytoplankton indicator species in tropical freshwater crater lakes in western Uganda (Tanttu et al. 2023). Using a combination of light and scanning electron microscopy, it was revealed that *Ulnaria acus* and *Fragilaria radians* were shown to have different spatiointerannual dynamics (Figure 3a,b). These two species growing in Lake Baikal are morphologically similar by light microscopy, but differ by SEM and rbcL and 18S rRNA (Zakharova et al. 2023).

The relative abundances of taxa obtained by metabarcoding are compositional data. In these data, many correlations between taxa may be artifactual, and true correlations may even appear with opposite signs (Friedman and Alm 2012). Spearman correlations between many taxa were detected for both relative and clr‐transformed relative abundance/biomass (Table E1: Appendix E). Correlations between dominant species and ASVs of diatoms (*Ulnaria acus*, *Aulacoseira islandica* , *Stephanodiscus meyeri*), Chrysophyceae ( *Dinobryon cylindricum* ), and Cryptophyceae (*Cryptomonas*) were detected for all metrics (Figure 4, Table E1: Appendix E). Previously, positive Spearman correlations were found between the relative abundance of phytoplankton species based on metabarcoding (ASVs of 16S rRNA or 23S rRNA belonging to the same species, grouped together) and microscopy for Bacillariophyta ( *Asterionella formosa* Hassall, *Diatoma tenuis* C. Agardh, *Ulnaria acus*) and Haptophyta ( *Chrysochromulina parva* ) in large alpine lakes in France (Lakes Aiguebelette, Annecy, Bourget, and Geneva; Gelis et al. 2024).

The relative abundance of dominant phytoplankton ASVs (Bacillariophyta, Chrysophyceae) was similar to the relative biomass of related phytoplankton species (Figure 1a,b). Correlations between the relative value of certain classes (Table 2) or species with ASVs (Table E1: Appendix E) were generally present for both abundance and biomass with minor differences in correlation coefficients. In general, relative and clr‐transformed relative abundance/biomass showed similar results, but in some cases contradictory. For example, for the classes Bacillariophyceae, Mediophyceae, Chrysophyceae, the correlation coefficients between relative abundance (meta) and biomass (mic) were slightly higher than with abundances (mic) (Table 2). However, for the same classes, the correlation coefficients for clr‐transformed relative abundance (meta) were slightly higher for abundances (mic) than for biomass (mic). Correlations between relative abundance (meta) of Cryptophyceae were higher for relative abundance (mic) than for biomass (mic), but no correlations were found for clr‐transformed relative values. Relative abundances of large‐cell phytoplankton Bacillariophyta and Dinophyceae based on ASVs of V4‐V5 18S rDNA showed better agreement with biovolume or carbon biomass than with microscopy abundance, while for small‐cell phytoplankton abundance showed a higher relationship (Andersson et al. 2023). A correlation was found between the dominant species *Stephanodiscus meyeri* and ASV12_Stephanodiscus for relative (*r* = 0.81) and absolute values (*r* = 0.86), but not for clr‐transformed relative values (Table E1: Appendix E). Correlations between relative or absolute abundance/biomass of *Cryptomonas gracilis* and ASV13_Cryptomonas were positive, but negative for clr‐transformed relative values. At the same time, a correlation between *Nitzschia graciliformis* and ASV32_Nitzschia was only found for clr‐transformed relative values and absolute values (ASV × class‐specific value). It is likely that small changes in the relative abundance of taxa become significant with clr‐transformation and therefore affect the results of the correlation analysis. It is possible that increasing the sample size will improve the result for the clr‐transformed relative values, and most likely more correlations will be consistent with the relative values.

Correlation analysis showed that microscopically identified Dinophyceae and Trebouxiophyceae had no correlations with the corresponding classes according to metabarcoding. Dinoflagellates have a high 18S rRNA gene copy number per cell, compared to Bacillariophyta and flagellated cells (Martin et al. 2022), which is likely to bias their relative abundance more than other classes, resulting in the lack of correlations with microscopically estimated abundance and biomass. For the relative abundance/biomass of the species *Fragilaria radians* , *Aulacoseira baicalensis*, *Gyrodinium helveticum*, *Gymnodinium baicalense*, *Koliella longiseta*, no correlations with related ASVs were found. The lack of correlations between related species and ASVs is due to the different dynamics of the taxa revealed by the different methods. The lack of ASV analogues for other phytoplankton species may be due to their low abundance and biomass in the samples, problems with DNA extraction from the cells of these species, or their lower affinity for the selected primers for amplification than other species. It has previously been shown that taxa of the Bacillariophyta, Charophyta, and Chlorophyta identified by microscopy were not detected by eDNA metabarcoding, which may be due to uncertainties associated with amplification and high‐throughput sequencing, and the incompleteness of the reference database (Hanžek et al. 2024).

Microbial community metabarcoding results can be affected by cross‐talk. A cross‐talk error (tag jumping) occurs when a read is assigned to an incorrect sample. On average, 2.6% and 2.1% of sequences had tag combinations that could be explained by tag jumping (Schnell, Bohmann, and Gilbert 2015). For eight datasets of 16S rRNA reads, cross‐talk rates are estimated to range from 0.4% to 1.5% mis‐assigned reads (Edgar 2018). In our work, we analyze the top 58 microeukaryotic ASVs (77%–95% of the total reads in the samples), from which we then extract the top 36 phytoplankton ASVs for correlation analysis with microscopy data. Therefore, in our dataset, the impact of possible cross‐talk on the results is extremely small.

Eustigmatophyceae and Dictyochophyceae, detected by metabarcoding alone, had high correlations with Chlorophyceae and Trebouxiophyceae, respectively. Due to the small size and morphological similarity of the cells belonging to species from these classes, it is unlikely that they can be accurately distinguished from each other. Therefore, Dictyochophyceae and Eustigmatophyceae are likely to be misidentified as Trebouxiophyceae or Chlorophyceae by microscopy. Combining the class‐specific relative abundance of phytoplankton taxa determined by metabarcoding with the class‐specific abundance and biomass estimated by microscopy will allow more accurate estimation of abundance and biomass for morphologically similar species.

### 4.2 Quantification of Metabarcoding Using Microscopy

In our work, absolute abundance/biomass of phytoplankton taxa (ASV) was calculated by combining relative abundances of ASVs with total or class‐specific abundance/biomass based on light microscopy. The accuracy of this quantification depends on the accuracy of the measured relative abundances of phytoplankton taxa estimated by metabarcoding and the quantitative parameters of phytoplankton by microscopy. Selected genes and any of its regions affect the relative abundance of phytoplankton taxa. Previously, relative abundances derived from 16S rRNA gene amplicon sequencing were combined with single‐cell enumeration using flow cytometry to quantify the absolute abundance of bacterial taxa (Props et al. 2017). Our approach is similar, but we have used primers that accurately represent the mean relative abundances of phytoplankton taxa. These primers were previously tested on microalgal mock communities (freshwater and marine microalgal species) with different relative abundances of taxa and then on environmental samples for validation (Bradley, Pinto, and Guest 2016). For quantification, it is better to use ASV produced by error‐correcting methods to reduce the number of errors in the sequences and identify a more accurate relative abundance of taxa. Error‐correcting methods used to analyze metabarcoding data estimate the composition and dynamics of taxa more accurately than methods that produce OTUs (Callahan et al. 2016; Edgar 2016). For example, in our previous work using primers from Bradley, Pinto, and Guest (2016) showed that ASV V8‐V9 18S rRNA of phytoplankton had a higher correlation with phytoplankton species biomass than ASV V4 18S rRNA, and OTUs (97% similarity) V4 and V8–V9 18S rRNA of phytoplankton during their seasonal dynamics in Lake Baikal (Bukin et al. 2023).

Coinertia analysis showed that the relationship between community structures was higher for absolute values than for relative values (Table 1). High correlations for all metrics (highest values for absolute values) were found in pairs (mic/meta) of the same classes Bacillariophyceae, Coscinodiscophyceae, Mediophyceae, Chrysophyceae, and Cryptophyceae. Chlorophyceae had correlations only for relative/absolute abundances. Prymnesiophyceae had correlations only for absolute abundance/biomass (Table 2). Many correlations between phytoplankton species and ASVs were found based on relative or absolute abundance/biomass, but for some species correlations were found based on absolute values only (*Lindavia baicalensis*, *Gymnodinium baicalense*, *Sphaeroсystis*; Table E1: Appendix E). Using the absolute abundance/biomass of ASVs estimated by both approaches (total or class‐specific), positive correlations with high coefficients were found with most dominant phytoplankton species. For example, both approaches revealed correlations for representatives of the classes Bacillariophyceae (*Ulnaria acus*/ASV2_Ulnaria_acus, *Nitzschia graciliformis* /ASV54_Fragilaria_vaucheriae), Coscinodiscophyceae ( *Aulacoseira islandica* /ASV9_Aulacoseira_islandica), Mediophyceae (*Stephanodiscus meyeri*/ASV12_Stephanodiscus, *Lindavia minuta*/ *L. baicalensis* /ASV52_Stephanodiscus), Chrysophyceae ( *Dinobryon cylindricum* / *D. cylindricum var. palustre* / *D. divergens* / *D. sociale* /ASV5_ *Dinobryon cylindricum* ), Dinophyceae (*Gymnodinium baicalense*/ASV23_Gymnodinium), Cryptophyceae (*Cryptomonas gracilis*/ASV13_Cryptomonas, *Komma caudata*/ASV13_Cryptomonas), Chlorophyceae (*Monoraphidium griffity*/ASV28_Choricystis_minor, Sphaeroсystis/ASV57_Chlamydomonadales), Prymnesiophyceae ( *Chrysochromulina parva* /ASV26_Chrysochromulina_parva; Table E1: Appendix E).

Each of the two approaches we used to quantify the absolute abundance/biomass of ASVs had its advantages and disadvantages. The advantage of the absolute abundance/biomass of ASVs based on the total abundance/biomass of phytoplankton (ASV × total value) is its simplicity of calculation. To calculate the absolute abundance of ASV × total value, it is only necessary to count the number of cells by microscopy without identifying them. For ASV × total value, there is no problem of zero values for certain samples, as the total abundance/biomass estimated by microscopy is present in all samples. Although ASV × total value is less accurate than ASV × class‐specific value for some parameters (lower correlation coefficients, not all species have correlations), its use revealed all correlations with dominant taxa as for ASV × class‐specific value (Table E1: Appendix E).

The advantage of the absolute abundance/biomass of ASVs based on the class‐specific abundance/biomass of phytoplankton (ASV × class‐specific value) is their higher agreement with absolute abundance/biomass of species (higher correlation coefficients) than ASV × total value (Table E1: Appendix E). This may be due to the fact that, in general, the cell sizes of species belonging to the same class are more similar to each other than the cell sizes of other classes. However, there are exceptions, such as *Ulnaria* and *Nitzschia*, which differ greatly in cell size. Correlations with specific species were only found for ASVs × class‐specific value, such as *Nitzschia graciliformis* /ASV32_Nitzschia, *Gyrodinium helveticum*/ASV8_Gyrodinium_helveticum, *Cryptomonas ovata* /ASV7_Cryptomonas curvata, *Rhodomonas pusilla*/ASV20_Plagioselmis_nannoplanctica, *Koliella longiseta*/ASV21_Chlorellales, and *Monoraphidium contortum* /ASV21_Chlorellales. The disadvantage of the ASV × class‐specific value is that it is not possible to calculate the absolute abundance/biomass for those samples in which ASV from a particular class had some relative abundance, but in the same samples microscopy did not reveal cells of species of the same class (0). In addition, microscopic identification of phytoplankton to class level by a qualified expert is required to calculate the ASV × class‐specific value.

Our results are consistent with the findings of studies on the spatiotemporal dynamics of spring phytoplankton in Lake Baikal assessed by light microscopy (Popovskaya et al. 2006, 2015; Pomazkina et al. 2010; Bondarenko et al. 2019). The use of absolute abundances/biomass estimated by metabarcoding in combination with microscopy allows a more accurate identification of differences in the structure of different phytoplankton communities and, as a consequence, will improve the assessment of the relationship between phytoplankton and environmental factors. For example, absolute phytoplankton metrics (abundance, biomass, biovolume) had a stronger relationship with river environmental variables than relative (percentage‐based) metrics (Reavie et al. 2010; Lavoie, Hamilton, and Poulin 2011). Thus, our proposed approach of combining metabarcoding and microscopy data, can be used to monitor phytoplankton in aquatic ecosystems alongside with the traditional use of microscopy alone.

## 5 Conclusions

Spatiointerannual dynamics of lake freshwater phytoplankton assessed by metabarcoding and microscopy were mostly consistent in relative and absolute abundances/biomasses of classes and genera/species. Differences in the identification and spatial distribution of some phytoplankton taxa show that each method has its limitations and advantages. For the quantification of phytoplankton metabarcoding, we propose the following steps: (a) use primers pretested on mock communities and natural samples that accurately estimate the relative abundance of taxa; (b) to improve the estimation of relative abundance of taxa, the resulting reads should be analyzed using bioinformatic methods that correct for PCR and sequencing errors; (c) to convert relative abundances of taxa into absolute abundance/biomass, use microscopy (morphology) data on total or class‐specific abundance/biomass of phytoplankton for the same samples. Comparison of the correlation coefficients between the relative or absolute abundances/biomasses of phytoplankton taxa estimated by both methods showed similar dynamics of dominant taxa for both quantitative metrics. This shows that the relative value of taxa estimated by metabarcoding remains a valid metric for assessing community structure. Quantification of absolute abundance and biomass of ASV phytoplankton using metabarcoding combined with microscopy allowed us to comprehensively characterize the spatiotemporal dynamics of phytoplankton in freshwater Lake Baikal, which can also be used for monitoring phytoplankton in other aquatic ecosystems.

## Author Contributions

**Ivan S. Mikhailov:** conceptualization (equal), formal analysis (equal), investigation (equal), methodology (equal), validation (equal), visualization (equal), writing – original draft (lead), writing – review and editing (lead). **Yurij S. Bukin:** conceptualization (equal), formal analysis (equal), investigation (equal), methodology (equal), software (lead), validation (equal), visualization (equal), writing – review and editing (supporting). **Alena D. Firsova:** formal analysis (supporting), investigation (supporting), writing – review and editing (supporting). **Darya P. Petrova:** investigation (supporting), writing – review and editing (supporting). **Yelena V. Likhoshway:** conceptualization (supporting), funding acquisition (lead), investigation (supporting), project administration (lead), supervision (lead), writing – review and editing (supporting).

## Conflicts of Interest

The authors declare no conflicts of interest.

## Data Availability

Raw sequence reads and metadata are deposited in the Sequence Read Archive—SRA (BioProject PRJNA1137590). Data and R scripts for data analysis is available on GitHub: https://github.com/bukinys/community_data_analysis.

## Appendix A

**TABLE A1:** Coordinates and dates of sampling in the southern basin of Lake Baikal for 3 years during spring.

Sample	Stations	Latitude	Longitude	Date
1_20	12 km from Kultuk	51 40.578	103 52.309	01.06.2020
2_20	3 km from Marituya	51 45.546	104 13.222	02.06.2020
3_20	Marituy‐Solzan	51 38.710	104 13.715	02.06.2020
4_20	3 km from Solzan	51 31.428	104 14.417	02.06.2020
5_20	Tolsty‐Snezhnaya	51 36.402	104 44.147	02.06.2020
6_20	3 km from Tankhoy	51 35.440	105 06.968	03.06.2020
7_20	Kadilny‐Mishikha	51 46.731	105 22.528	03.06.2020
8_20	Listvyanka‐Tankhoy	51 42.262	105 00.720	03.06.2020
9_20	3 km from Listvyanka	51 49.033	104 54.616	03.06.2020
1_21	12 km from Kultuk	51 40.578	103 52.309	02.06.2021
2_21	3 km from Marituya	51 45.546	104 13.222	02.06.2021
3_21	Marituy‐Solzan	51 38.710	104 13.715	02.06.2021
4_21	3 km from Solzan	51 31.428	104 14.417	03.06.2021
5_21	Tolsty‐Snezhnaya	51 36.402	104 44.147	03.06.2021
6_21	3 km from Tankhoy	51 35.440	105 06.968	04.06.2021
7_21	Kadilny‐Mishikha	51 46.731	105 22.528	04.06.2021
8_21	Listvyanka‐Tankhoy	51 42.262	105 00.720	03.06.2021
9_21	3 km from Listvyanka	51 49.033	104 54.616	03.06.2021
1_22	12 km from Kultuk	51 40.578	103 52.309	02.06.2022
2_22	3 km from Marituya	51 45.546	104 13.222	02.06.2022
3_22	Marituy‐Solzan	51 38.710	104 13.715	02.06.2022
4_22	3 km from Solzan	51 31.428	104 14.417	02.06.2022
5_22	Tolsty‐Snezhnaya	51 36.402	104 44.147	03.06.2022
6_22	3 km from Tankhoy	51 35.440	105 06.968	03.06.2022
7_22	Kadilny‐Mishikha	51 46.731	105 22.528	04.06.2022
8_22	Listvyanka‐Tankhoy	51 42.262	105 00.720	03.06.2022
9_22	3 km from Listvyanka	51 49.033	104 54.616	03.06.2022

## Appendix B

**TABLE B1:** The number of reads, species richness and diversity for ASVs of microeukaryotes in the south basin of Lake Baikal for 3 years during spring.

Sample	Stations	Reads	Richness	Chao1	ACE	Shannon	Simpson
1_20	S12K_1	73,157	222	227	223	3.08	0.87
2_20	S3M_1	57,382	185	192	187	2.77	0.79
3_20	SMS_1	87,319	217	224	219	3.21	0.89
4_20	S3S_1	60,896	190	190	190	3.52	0.92
5_20	STS_1	16,611	117	117	117	3.29	0.91
6_20	S3T_1	82,910	277	278	278	3.34	0.90
7_20	SKM_1	37,366	189	189	189	3.48	0.92
8_20	SLT_1	33,117	147	152	149	3.38	0.91
9_20	S3L_1	13,993	90	90	90	2.97	0.87
1_21	S12K_2	55,995	165	168	167	3.04	0.90
2_21	S3M_2	90,775	225	226	225	2.93	0.88
3_21	SMS_2	33,735	117	127	118	2.61	0.85
4_21	S3S_2	34,469	115	115	115	2.51	0.83
5_21	STS_2	36,687	114	114	114	2.32	0.74
6_21	S3T_2	73,582	194	210	199	2.59	0.77
7_21	SKM_2	82,787	175	175	175	2.50	0.77
8_21	SLT_2	81,391	213	217	215	2.75	0.82
9_21	S3L_2	98,176	226	229	227	2.81	0.84
1_22	S12K_3	36,171	158	159	158	3.47	0.92
2_22	S3M_3	41,860	141	142	142	2.62	0.81
3_22	SMS_3	27,127	122	122	122	2.90	0.84
4_22	S3S_3	57,030	203	206	204	3.53	0.93
5_22	STS_3	34,700	156	163	159	3.77	0.96
6_22	S3T_3	46,742	213	214	214	3.63	0.92
7_22	SKM_3	24,686	157	159	158	3.33	0.87
8_22	SLT_3	25,566	166	166	167	3.65	0.92
9_22	S3L_3	31,918	201	201	201	3.91	0.95

## Appendix D

**TABLE D1:** Mean richness of ASV and species of different phytoplankton classes, and Spearman correlations between ASVs and species number of different phytoplankton classes.

Сlass_names	Mean_richness_ASV	Mean_richness_species	*r*_value	*p*
Bacillariophyceae	3.6	3.0	0.18	0.367
Coscinodiscophyceae	2.7	1.5	0.19	0.319
**Mediophyceae**	1.9	2.2	**0.52**	**0.005**
Chrysophyceae	20.3	4.2	−0.35	0.072
Dinophyceae	7.4	2.3	−0.05	0.804
**Cryptophyceae**	10.4	2.6	**−0.46**	**0.015**
Trebouxiophyceae	5.1	0.8	0.34	0.083
**Chlorophyceae**	2.5	3.1	**0.49**	**0.008**
Prymnesiophyceae	2.3	0.5	−0.12	0.549

*Note:* Classes with significant correlation coefficients are shown in bold.

**TABLE D2:** Abundance, species richness and diversity for 23 phytoplankton species and 1 group (stomatocysts) in the southern basin of Lake Baikal for 3 years during spring.

Sample	Stations	Abundance	Richness	Chao1	ACE	Shannon	Simpson
1_20	S12K_1	123,540	12	12	—	1.65	0.76
2_20	S3M_1	103,980	11	11	—	1.68	0.77
3_20	SMS_1	77,210	12	12	—	1.76	0.79
4_20	S3S_1	111,360	15	15	—	2.09	0.84
5_20	STS_1	95,390	13	13	—	1.92	0.82
6_20	S3T_1	110,000	12	12	—	1.55	0.68
7_20	SKM_1	63,080	12	12	—	1.54	0.69
8_20	SLT_1	43,210	13	13	—	1.88	0.76
9_20	S3L_1	46,740	10	10	—	1.43	0.68
1_21	S12K_2	258,580	15	15	—	1.48	0.66
2_21	S3M_2	97,790	15	15	—	1.39	0.54
3_21	SMS_2	56,600	17	17	—	1.94	0.74
4_21	S3S_2	194,330	20	20	—	2.01	0.76
5_21	STS_2	870,200	19	19	—	1.82	0.77
6_21	S3T_2	521,810	18	18	—	1.91	0.79
7_21	SKM_2	569,640	21	21	—	1.60	0.67
8_21	SLT_2	681,140	19	19	—	1.16	0.47
9_21	S3L_2	526,660	15	15	—	1.28	0.58
1_22	S12K_3	188,680	16	16	—	1.97	0.81
2_22	S3M_3	181,500	15	15	—	2.03	0.82
3_22	SMS_3	268,990	17	17	—	2.07	0.80
4_22	S3S_3	226,900	18	18	—	1.86	0.75
5_22	STS_3	33,370	19	19	—	1.84	0.75
6_22	S3T_3	523,200	19	19	—	1.75	0.72
7_22	SKM_3	251,190	15	15	—	0.87	0.35
8_22	SLT_3	243,500	17	17	—	1.10	0.44
9_22	S3L_3	194,090	13	13	—	1.10	0.46

**TABLE D3:** Number of reads, species richness and diversity for the top 36 ASVs in the southern basin of Lake Baikal for 3 years during spring.

Sample	Stations	Number of reads	Richness	Chao1	ACE	Shannon	Simpson
1_20	S12K_1	42,509	30	30	—	1.98	0.72
2_20	S3M_1	39,493	27	27	—	1.61	0.59
3_20	SMS_1	49,596	27	27	—	1.89	0.71
4_20	S3S_1	34,404	31	31	—	2.22	0.808
5_20	STS_1	9183	23	23	—	2.17	0.78
6_20	S3T_1	45,443	28	28	—	2.13	0.76
7_20	SKM_1	19,187	29	29	—	2.48	0.84
8_20	SLT_1	17,703	27	27	—	2.26	0.78
9_20	S3L_1	8205	23	23	—	1.98	0.73
1_21	S12K_2	33,162	31	31	—	2.07	0.79
2_21	S3M_2	44,536	30	30	—	2.07	0.78
3_21	SMS_2	16,367	27	27	—	1.90	0.74
4_21	S3S_2	14,967	29	29	—	1.82	0.69
5_21	STS_2	11,628	27	27	—	2.45	0.87
6_21	S3T_2	23,655	34	34	—	2.64	0.89
7_21	SKM_2	30,278	33	33	—	2.48	0.87
8_21	SLT_2	31,701	32	32	—	2.36	0.83
9_21	S3L_2	45,703	32	32	—	2.29	0.82
1_22	S12K_3	19,096	31	31	—	2.86	0.92
2_22	S3M_3	10,684	30	30	—	2.95	0.93
3_22	SMS_3	9517	27	27	—	2.77	0.90
4_22	S3S_3	21,804	31	31	—	2.71	0.88
5_22	STS_3	21,705	31	31	—	2.79	0.91
6_22	S3T_3	32,909	34	34	—	2.50	0.85
7_22	SKM_3	17,815	31	31	—	2.23	0.76
8_22	SLT_3	16,957	31	31	—	2.48	0.83
9_22	S3L_3	19,292	33	33	—	2.68	0.88

## Appendix E

**TABLE E1:** Spearman correlation coefficients between different quantitative data of related phytoplankton species (microscopy) and ASVs (metabarcoding).

Pairs of correlating taxa		Relative values				Absolute values			
Species	ASVs	Nontransformed		Clr‐transformed		ASV × Total value		ASV × Class‐specific value	
		Abundance (species)/abundance (ASV)	Biomass (species)/abundance (ASV)	Abundance (species)/abundance (ASV)	Biomass (species)/abundance (ASV)	Abundance (species)/abundance (ASV)	Biomass (species)/biomass (ASV)	Abundance (species)/abundance (ASV)	Biomass (species)/biomass (ASV)
*Ulnaria acus* (Kützing) Aboal	ASV2_Ulnaria_acus	0.7	0.73	0.7	0.68	0.65	0.73	0.75	0.89
*Nitzschia graciliformis* Lange‐Bertalot & Simonsen	ASV32_Nitzschia	—	—	0.77	0.83	—	—	0.38	0.38
	ASV54_Fragilaria_vaucheriae	0.44	0.39	0.66	0.69	0.47	0.47	0.49	0.49
*Aulacoseira islandica* (O. Müller) Simonsen	ASV9_Aulacoseira_ islandica	0.38	0.4	0.66	0.65	0.8	0.81	0.88	0.8
	ASV12_Stephanodiscus	0.52	0.55	—	—	0.55	0.55	0.49	0.49
	ASV52_Stephanodiscus	—	—	0.48	0.41	—	—	—	—
*Stephanodiscus meyeri* Genkal & Popovskaya	ASV12_Stephanodiscus	0.81	0.81	—	—	0.86	0.86	0.9	0.9
	ASV52_Stephanodiscus	—	—	0.39	0.39	0.41	0.39	0.59	0.54
*Lindavia minuta* (Skvortzov) T. Nakov & al.	ASV52_Stephanodiscus	0.58	0.55	—	—	0.59	0.71	0.57	0.57
*Lindavia baicalensis* (Skvortsov & K.I. Meyer) Nakov, Guillory, M.L. Julius, E.C. Theriot & A.J. Alverson	ASV52_Stephanodiscus	—	—	—	—	0.54	0.38	0.54	0.6
*Dinobryon cylindricum* O.E. Imhof	ASV5_ *Dinobryon cylindricum*	0.63	0.72	0.64	0.58	0.8	0.81	0.83	0.83
*Dinobryon cylindricum var. palustre* Lemmermann	ASV5_ *Dinobryon cylindricum*	0.64	0.64	0.39	0.46	0.62	0.58	0.63	0.59
*Dinobryon divergens* O.E. Imhof	ASV5_ *Dinobryon cylindricum*	0.65	0.67	—	—	0.6	0.61	0.58	0.57
	ASV59_Hydrurales	—	—	—	0.39	—	—	—	—
*Dinobryon sociale* (Ehrenberg) Ehrenberg	ASV5_ *Dinobryon cylindricum*	0.52	0.55	—	—	0.55	0.61	0.58	0.56
Stomatocysts	ASV22_Chrysophyceae	0.5	0.49	—	—	—	—	—	—
*Gymnodinium baicalense* N.L. Antipova	ASV23_Gymnodinium	—	—	—	—	0.54	0.55	0.63	0.66
*Gyrodinium helveticum* (Penard) Y. Takano & T. Horiguchi	ASV8_Gyrodinium_ helveticum	—	—	—	—	—	—	0.9	0.84
*Cryptomonas ovata* Ehrenberg	ASV7_Cryptomonas curvata	0.42	0.44	—	0.49	—	—	0.62	0.73
	ASV13_Cryptomonas	—	—	0.45	—	—	—	—	0.4
*Cryptomonas gracilis* Skuja	ASV13_Cryptomonas	0.49	0.5	−0.45	—	0.71	0.74	0.69	0.7
	ASV14_Cryptophyceae	−0.47	−0.48	0.38	—	—	—	—	—
	ASV48_Cryptomonadales	—	—	—	0.41	0.62	0.57	0.48	0.49
*Rhodomonas pusilla* (Bachmann) Javornický	ASV20_Plagioselmis_ nannoplanctica	0.47	0.44	—	—	—	—	0.76	0.62
	ASV34_Chroomonas	—	—	—	—	0.5	—	0.81	0.62
*Komma caudata* (L. Geitler) D.R. A.Hill	ASV13_Cryptomonas	0.43	—	—	—	0.62	0.6	0.58	0.55
	ASV34_Chroomonas	—	—	—	—	0.62	—	0.51	0.43
*Koliella longiseta* (Vischer) Hindák	ASV21_Chlorellales	—	—	—	—	—	—	0.74	0.75
*Monoraphidium contortum* (Thuret) Komárková‐Legnerová	ASV21_Chlorellales	0.4	—	0.43	—	—	—	0.58	0.57
*Monoraphidium griffithii* (Berkeley) Komárková‐Legnerová	ASV21_Chlorellales	—	—	0.43	—	0.52	0.53	—	—
	ASV28_Choricystis_ minor	—	—	0.38	—	0.48	0.5	0.39	0.46
*Sphaeroсystis* sp.	ASV57_Chlamydomonadales	—	—	—	—	0.56	0.54	0.42	—
*Chrysochromulina parva* Lackey	ASV26_Chrysochromulina_parva	—	—	0.39	—	0.74	0.47	1	1

*Note:* The reliable value (*p* ≤ 0.05) of the correlation coefficient is *r* ≥ |0.38|.
